# Erysense, a Lab-on-a-Chip-Based Point-of-Care Device to Evaluate Red Blood Cell Flow Properties With Multiple Clinical Applications

**DOI:** 10.3389/fphys.2022.884690

**Published:** 2022-04-27

**Authors:** Steffen M. Recktenwald, Marcelle G. M. Lopes, Stephana Peter, Sebastian Hof, Greta Simionato, Kevin Peikert, Andreas Hermann, Adrian Danek, Kai van Bentum, Hermann Eichler, Christian Wagner, Stephan Quint, Lars Kaestner

**Affiliations:** ^1^ Experimental Physics, Saarland University, Saarbruecken, Germany; ^2^ Cysmic GmbH, Saarbruecken, Germany; ^3^ Theoretical Medicine and Biosciences, Saarland University, Saarbruecken, Germany; ^4^ Institute for Clinical and Experimental Surgery, Saarland University, Campus University Hospital, Homburg, Germany; ^5^ Translational Neurodegeneration Section “Albrecht-Kossel”, Department of Neurology, University Medical Center Rostock, University of Rostock, Rostock, Germany; ^6^ DZNE, Deutsches Zentrum für Neurodegenerative Erkrankungen, Research Site Rostock/Greifswald, Rostock, Germany; ^7^ Center for Transdisciplinary Neurosciences Rostock (CTNR), University Medical Center Rostock, University of Rostock, Rostock, Germany; ^8^ Neurologische Klinik und Poliklinik, Ludwig-Maximilians-University, Munich, Germany; ^9^ MVZ Saarpfalz, Homburg, Germany; ^10^ Institute for Clinical Hemostaseology and Transfusion Medicine, Saarland University and Saarland University Hospital, Homburg, Germany; ^11^ Department of Physics and Materials Science, University of Luxembourg, Luxembourg City, Luxembourg

**Keywords:** erythrocyte, microfluidics, shape classification, artificial capillary, neuroacanthocytosis syndrome, hemodiafiltration, red cell storage, phase diagram

## Abstract

In many medical disciplines, red blood cells are discovered to be biomarkers since they “experience” various conditions in basically all organs of the body. Classical examples are diabetes and hypercholesterolemia. However, recently the red blood cell distribution width (RDW), is often referred to, as an unspecific parameter/marker (e.g., for cardiac events or in oncological studies). The measurement of RDW requires venous blood samples to perform the complete blood cell count (CBC). Here, we introduce Erysense, a lab-on-a-chip-based point-of-care device, to evaluate red blood cell flow properties. The capillary chip technology in combination with algorithms based on artificial neural networks allows the detection of very subtle changes in the red blood cell morphology. This flow-based method closely resembles *in vivo* conditions and blood sample volumes in the sub-microliter range are sufficient. We provide clinical examples for potential applications of Erysense as a diagnostic tool [here: neuroacanthocytosis syndromes (NAS)] and as cellular quality control for red blood cells [here: hemodiafiltration (HDF) and erythrocyte concentrate (EC) storage]. Due to the wide range of the applicable flow velocities (0.1–10 mm/s) different mechanical properties of the red blood cells can be addressed with Erysense providing the opportunity for differential diagnosis/judgments. Due to these versatile properties, we anticipate the value of Erysense for further diagnostic, prognostic, and theragnostic applications including but not limited to diabetes, iron deficiency, COVID-19, rheumatism, various red blood cell disorders and anemia, as well as inflammation-based diseases including sepsis.

## Introduction

Most diagnostic tests target one particular medical question that arises from the medical history and clinical examination of a general or specialized physician. Quite often, these diagnostic measures are expressed as numeric values. However, such singularities often do not reflect the pathology of a patient, and multiple tests need to be performed to obtain a conclusive picture. Furthermore, most blood-derived diagnostic or scientific parameters are assessed in stasis or even on dead cells, *e.g.*, peripheral blood smear (McPhedran and Hall, 2005), erythrocyte sedimentation rate (Darras et al., 2021), electron microscopy (Bessis, 1974), ion flux measurements (Bernhardt and Ellory, 2003). In contrast, blood *in vivo* is under permanent flow, while blood in stasis is not compatible with life.

Here, we present a red blood cell (RBC) diagnostic approach that is, based on the dynamic shape changes in the capillary *in vitro* flow (Kihm et al., 2018). The (3D) shape of RBCs is a highly sensitive parameter already in stasis (Simionato et al., 2021) with increased information content in capillary flow coming along with a diagnostic potential (Brust et al., 2014; Quint et al., 2017; Rabe et al., 2021).

Our approach differs from numerous microfluidic investigations of RBCs by the combination of three main properties: (i) we use in contrast to many other studies (Iragorri et al., 2018; Rizzuto et al., 2021) straight channels without any constrictions; (ii) we use a micro-capillary size (8 μm × 11 µm diameter), whereas others use dimensions in the range of 30–50 µm (D’Apolito et al., 2016; Inglebert et al., 2020); and (iii) we use flow velocities in the physiological range well below the flow velocity of other commercial devices, such as the AcCellerator by Zellmechanik Dresden (Germany), (Reichel et al., 2019).

During flow, in our approach, both, intrinsic properties and external influences are reflected in the RBC shape. The intrinsic parameters include the viscosity of the cytoplasm, properties of the cytoskeleton, membrane permeability, or stiffness. External conditions are besides the channel geometry and the applied pressure drop (flow velocity), chemical mediators such as amino acids, hormones, cytokines, or drugs. Since RBCs constantly circulate throughout the entire body, they can sense all kinds of abnormalities. Therefore, we provide examples supporting the concept that RBCs can be used as a widely applicable biomarker for undiagnosed or difficult-to-diagnose diseases, known diseases, patient-therapy monitoring or treatment tests *in vitro*. Furthermore, we provide a test for the functionality of stored RBCs prior to transfusions, an aspect that is, so far largely ignored due to the lack of appropriate testing methods.

## Materials and Methods

### Blood Sample Preparation

Blood sample collection was approved by the “Ärztekammer des Saarlandes,” ethics permission 51/18, and performed after informed consent was obtained according to the declaration of Helsinki. Blood samples from neuroacanthocytosis syndrome (NAS) patients [chorea acanthocytosis/VSP13A disease (ChAc) or McLeod Syndrome (MLS)] were taken by venous puncture and measured within 2–8 h from the withdrawal. The diagnosis of ChAc and MLS was based on the clinical phenotype and has been confirmed by detection of the *VPS13A* mutation and/or absence of the chorein in RBC membranes via western blot (ChAc) or the detection of *XK* mutations and/or the immunehematological determination of McLeod blood phenotype (MLS) (Peikert et al., 2022). Further NAS patient information is summarized in Supplementary Table S1 in the Supplementary Material.

Blood of dialysis patients was collected before and immediately after hemodiafiltration with CorDiax (Fresenius Medical Care, Germany) in lithium heparin tubes. Patients requiring regular dialysis were investigated after a weekend, i.e., the longest period in the week without dialysis. Hemodiafiltration (HDF) took approximately 4–5 h (for patient information refer to Supplementary Table S2 in the Supplemental Material).

For the investigation of stored RBCs, we tested samples of erythrocyte concentrates (EC) with additive solution [phosphate–adenine–glucose–guanosine–saline–mannitol (PAGGSM)] for clinical use. The RBCs samples were generated at defined time points of 4°C storage by careful slewing of the storage bag followed by stripping a firmly attached tube segment with a roller clamp. This technique enabled sampling of a representative aliquot of the EC.

Blood samples were suspended in phosphate-buffered saline solution (PBS, Gibco, Schwerte, Germany). The sample was centrifuged for 5 minutes at 1,500 × g to separate the RBCs from plasma, leukocytes, and platelets. Subsequently, sedimented RBCs were resuspended in PBS, and the centrifugation and washing steps were repeated three times. Finally, a hematocrit of 0.5% was adjusted in a PBS solution that contained 1 g/L bovine serum albumin (BSA, Sigma-Aldrich; Taufkirchen, Germany).

For the dialysis patients, RBCs were suspended in autologous plasma, whereas RBC and plasma were separated as described above. In addition, the plasma was further centrifuged at 5000 × g for 5 min to assure the removal of all leukocytes and platelets. Subsequently, the top plasma layer was used as the measurement fluid.

### Microfluidic Set-Up

**FIGURE 1:**
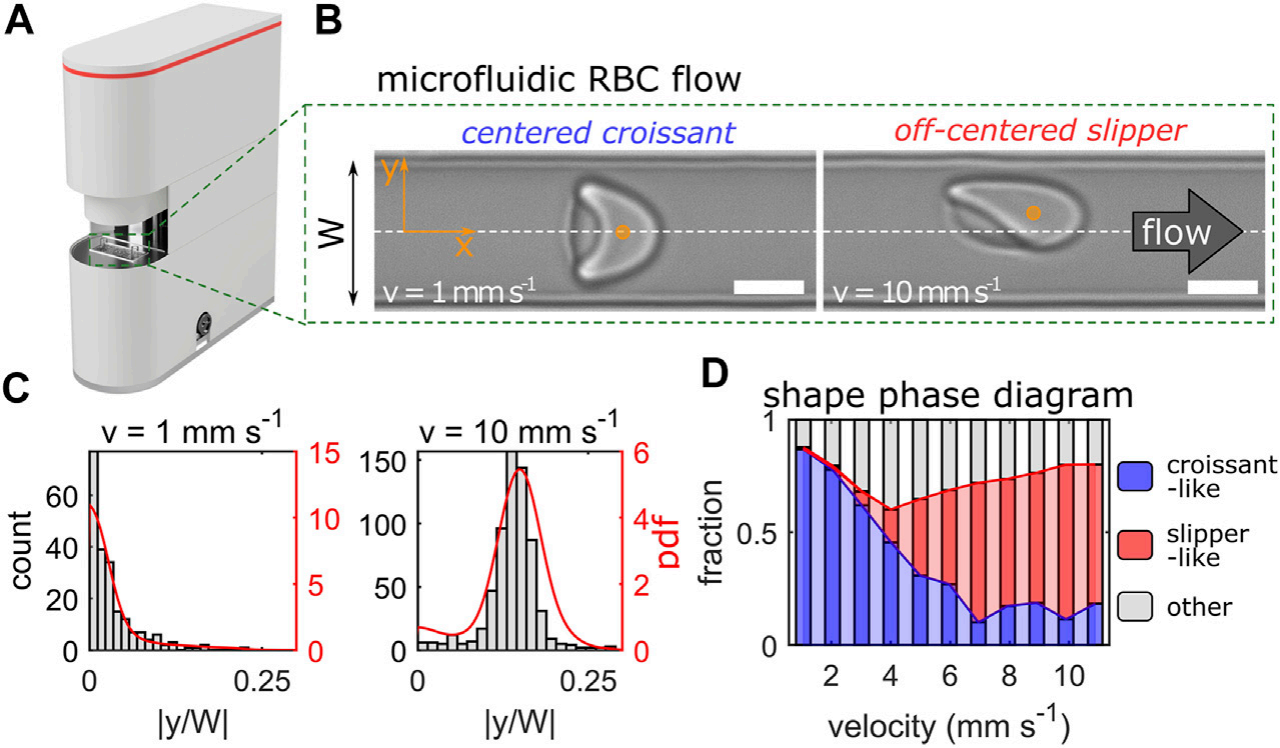
Erysense® device and principle of measurement. **(A)** Image of the Erysense® device. **(B)** Representative images of a croissant-shaped and a slipper-shaped RBC at low (1 mm/s) and high (10 mm/s) velocity, respectively. Scale bars represent 5 µm. Flow is from left to right and the dashed white line indicates the channel centerline in the *y*-direction with a channel width W. **(C)** Representative histograms and probability density functions (pdf) of the normalized cell’s center-of-mass in *y*-direction at a low (1 mm/s) and high (10 mm/s) velocity. **(D)** Representative shape phase diagram of a healthy control showing croissant-like, slipper-like, and other RBC shapes as a function of the cell velocity.

The Erysense® device (Cysmic, Saarbruecken, Germany) (Figure 1A) employs a microfluidic chip with parallel microcapillaries, which are fabricated using polydimethylsiloxane (PDMS) through standard soft lithography (Friend and Yeo, 2010). The microfluidic channels have a rectangular cross-section with a height of 8 μm, a width of 11 μm, and a total length of 40 mm. A high-precision pressure device is used to apply a constant pressure drop in a range of 100 mbar to 1 bar and pump the RBC suspension through the microfluidic chip.

RBC flow is recorded with a USB 3.0 camera (DMK 23U1300, The Imaging Source; Bremen, Germany). The camera is aligned along the channel’s *z*-direction, hence the flow in the *x-y*-plane is imaged (Figure 1B). Single-cell flow is recorded with a frame rate of up to 400 Hz depending on the applied pressure drop and subsequently processed.

### Data Analysis

Each frame of the image sequence was analyzed with a custom python script to detect passing cells. For each RBC, the cell’s 2D-center-of-mass in the *x-y*-plane was determined (schematically highlighted as orange dots in Figure 1B) and the cell velocity was calculated by tracking its position over the image sequence within the field of view. Based on the velocity of the RBCs, the distribution of the absolute value of the cell’s y-position, normalized by the channel width W, was determined. Figure 1C shows two histograms and the corresponding probability density distributions (pdfs) for control RBCs with mean velocities of 1 mm/s and 10 mm/s. At low velocities, RBCs preferentially form axisymmetric croissants that flow in the channel center at |y/W| = 0, while slipper-shaped RBCs dominate at higher velocities, which exhibit an off-centered equilibrium position at |y/W| > 0. The distribution of the RBC lateral *y*-position for a given velocity is a characteristic indicator of the single-cell flow in such confined microchannels (Guckenberger et al., 2018; Kihm et al., 2018; Recktenwald et al., 2022) and was used as a reference point for pathological changes in RBC flow behavior at specific cell velocities. Data processing was roughly half as fast as the acquisition and started almost simultaneously (but scales with CPU power).

To check for significant differences, a one-way ANOVA test with Tukey’s multiple comparison was performed.

### RBC Classification Using a Convolutional Neural Network

To enable a fast and automated classification of RBC shapes, we used a convolutional neural network (CNN), as described previously (Kihm et al., 2018). The CNN consists of an image input layer, several subsequent convolution stages, and an output layer. Details about the combination of convolution, rectification, and max-pooling are summarized in Supplementary Table S3 in the Supplementary Material. We employed a supervised training of the CNN according to (Kihm et al., 2018). Our training data set consisted of seven different classes. Besides the characteristic croissant and slipper shapes that dominate healthy RBC flow in microchannels (Figure 1B) (Guckenberger et al., 2018; Recktenwald et al., 2022), complimentary pathological classes that exhibit pathophysiologic RBC shapes were identified. A representative overview of all RBC shape classes is provided in Supplementary Figure S1 of the Supplementary Material. Further, we define the so-called shape ratio as the proportion of pathophysiologic to healthy RBC shapes. Hence, for a healthy control, the shape ratio is considerably smaller than one. A summary of the RBC shapes for a given sample is the so-called RBC shape phase diagram, *i.e.*, the frequency of occurrence of RBC shapes as a function of their velocity. Figure 1D shows the phase diagram for a healthy control sample. Here, croissant-like RBC shapes dominate at lower velocities, while slipper-shaped RBCs predominantly emerge above velocities of 6 mm/s, in accordance with previous experimental and numerical studies on similar channels (Guckenberger et al., 2018; Recktenwald et al., 2022).

## Results

### Design of the Device

The device we introduce (Erysense, Figure 1) employs a microfluidic chip that mimics capillary size (8 μm × 11 µm) similar to *in vivo*. Thus, the method needs only a small quantity of RBCs (<1 µL), *e.g.*, via needle finger prick. The RBC flow can be changed in terms of flow velocity and/or the ambient solution. Applying a pressure drop between 100 mbar and 1 bar allows us to examine the RBC flow properties in a broad velocity range of 0.1–10 mm/s, depending on the surrounding fluid. Similar velocities are also found in the microvascular network (Pries et al., 1995; Secomb, 2017). These external conditions induce very subtle changes in the cellular morphology, resulting in a certain cell shape distribution according to the pressure drop applied. While similar approaches were applied in the context of basic research, *e.g.*, (Brust et al., 2014; Danielczok et al., 2017; Kihm et al., 2018; Rabe et al., 2021) rather reflecting a “chip-in-the-lab” scenario, we introduce here a compact table-top device with microfluidic chips produced with industrial standards implementing the “lab-on-a-chip” concept.

### Diagnostic Parameter for the Neuroacanthocytosis Syndrome

**FIGURE 2:**
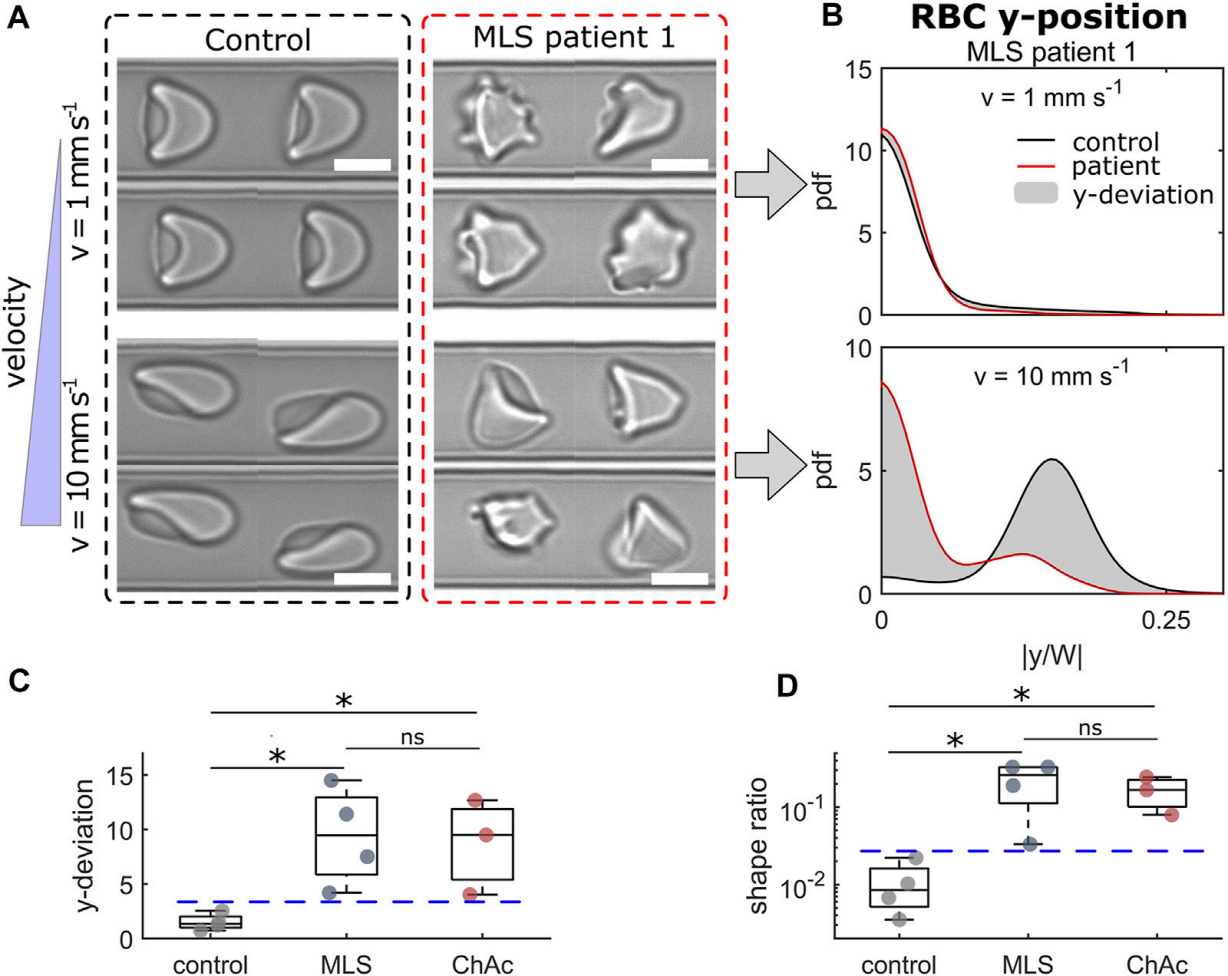
Results of RBCs from neuroacanthocytosis syndrome (NAS) patients. **(A)** Representative RBC shapes for a healthy control (left) and an MLS patient (right) at low (top) and high velocity (bottom). Scale bars represent 5 µm. **(B)** Probability density functions (pdfs) of the cell’s y-position distribution for the control and an MLS patient at the velocities shown in **(A)**. The gray area indicates the difference between both distributions. **(C)** Deviation between the y-position distributions based on the average distribution for healthy controls in the entire velocity range of 1–10 mm/s. **(D)** Shape ratio between non-normal RBCs (acanthocytes and other pathological shapes) and normocytes. * refers to a significance level of *p* < 0.05 and ns stands for not significant. Blue dashed horizontal lines represent thresholds between controls and NAS patients. The analysis presented was performed on an average of 4519 cells per patient/donor (between 2422 and 5484 cells).

The NAS subsumes the diseases ChAc (Peikert et al., 2017) and MLS (Jung et al., 2011). Both are rare progressive neurodegenerative disorders characterized by neurological symptoms such as movement disorders (chorea, parkinsonism, dystonia), epileptic seizures, cognitive impairment and the presence of acanthocytes. ChAc usually manifests earlier in life (in the twenties) than MLS (often after 40 years of age). Diagnosis is complicated, often delayed, and needs to be confirmed by genetic testing (*VPS13A* gene in ChAc, *XK* gene in MLS) and chorein in western blot (ChAc) or immunohaematological assessments (MLS) (Peikert et al., 2022). Acanthocyte detection relies on a wet unfixed blood smear, which is not a generally available diagnostic method (Storch et al., 2005). The erythrocyte sedimentation rate (ESR) is slower in NAS patients compared to healthy control subjects and was hence recently proposed as an additional diagnostic marker (Darras et al., 2021). Figure 2A shows representative RBC shapes of a healthy control and an MLS patient at two velocities. At 1 mm/s, we mainly observed croissant-shaped cells for the control, while the MLS patient showed a pronounced amount of acanthocytes. Since both croissants and acanthocytes preferentially flow in the channel center at low velocities, the probability density functions (pdfs) of their y-positions are very similar, as shown in the top panel of Figure 2B. However, when increasing the flow velocity, healthy control RBCs predominantly formed off-centered slippers, while patient RBCs did not deform into stable off-centered shapes, but preferentially flowed in the mid of the channel. Hence, we observed a strong deviation between the *y*-distributions of both groups, as indicated by the gray area in the lower panel of Figure 2B. The sum of this deviation from the average overall controls is shown in Figure 2C for a velocity range of 1–10 mm/s. Figure 2D shows the shape ratio between pathological RBCs shapes (including acanthocytes) to normocytes. For both parameters (Figures 2C,D), we revealed good discrimination between RBCs of healthy donors and NAS patients.

### Comparison of Red Blood Cells Before and After Hemodiafiltration

During the past decade, the number of patients with kidney failure has increased dramatically (Thurlow et al., 2021) and different dialysis modalities are applied. However, the effect of dialysis procedures on RBCs is still not fully understood. We face the situation that both renal failure (Ertan et al., 2017) and the dialysis procedure (Irino et al., 2019) impair RBC properties. In particular, hemodiafiltration (HDF) was reported to show (temporary) adverse effects on RBCs (Georgatzakou et al., 2018). However, selection and optimization processes require fast and predictive tests of the RBCs.

**FIGURE 3:**
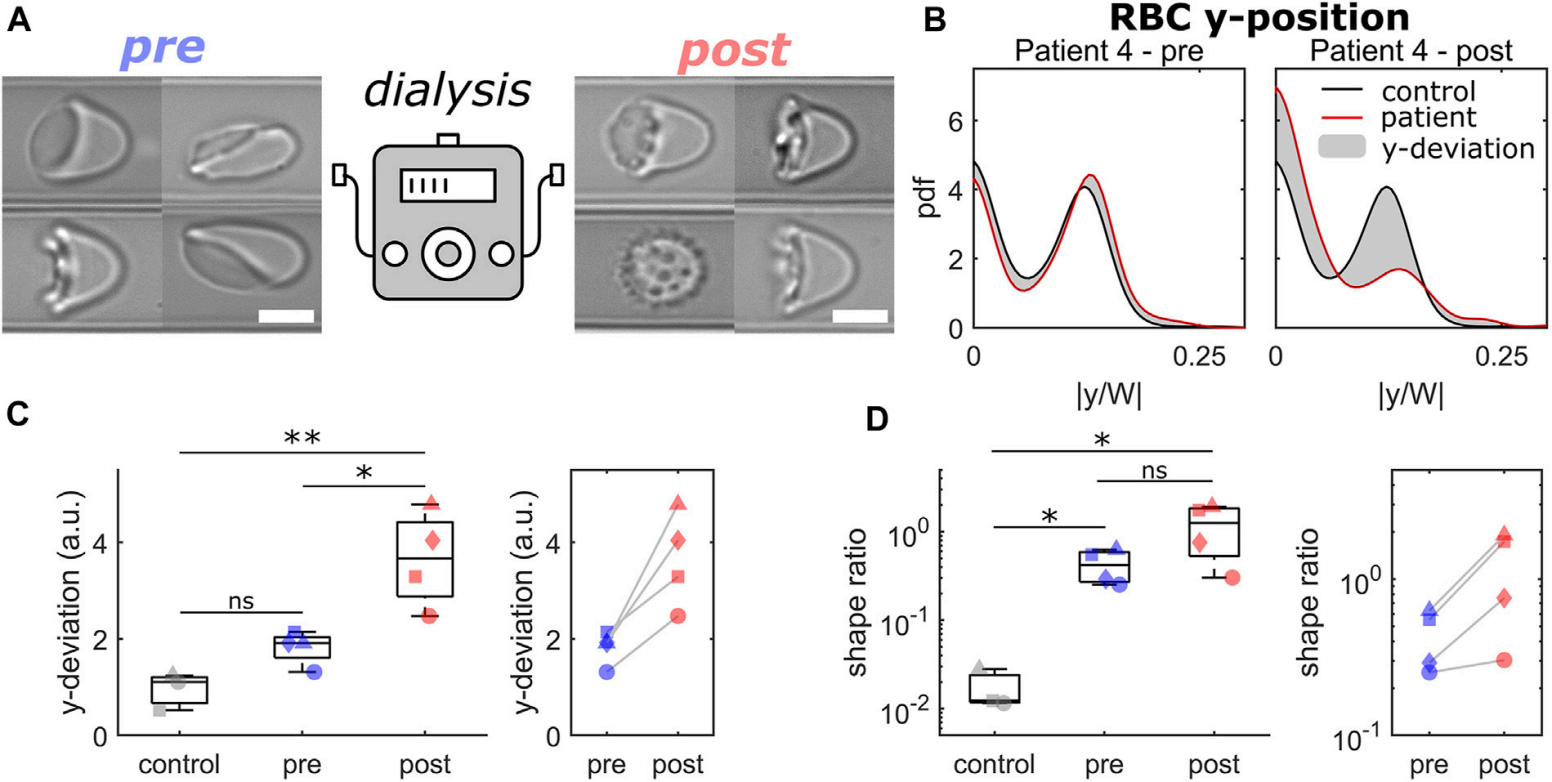
Comparison of RBCs from patients before and after dialysis by hemodiafiltration. **(A)** Representative RBC shapes pre (left) and post (right) hemodiafiltration. Scale bars represent 5 µm. **(B)** Probability density functions (pdfs) of the normalized cell’s center-of-mass position at v = 5 mm/s. Black and red lines represent the pdfs for a healthy control and for patient 4 pre (left) and (post) hemodiafiltration. The gray area indicates the difference between both pdfs. **(C)** Deviation between the y-position distributions based on the average distribution for healthy controls for a velocity range of 1–10 mm/s. **(D)** Shape ratio between pathological and healthy RBC shapes in the velocity range of 1–3 mm/s. Data shown with the same symbols in **(C)** and **(D)** correspond to the same patient pre and post hemodiafiltration. * refers to a significance level of *p* < 0.05, ** to *p* < 0.01, and ns stands for not significant. The analysis presented was performed on an average of 4006 cells per patient/donor (between 2895 and 5496 cells).

Here, we investigated blood samples of 4 patients before and immediately after HDF for their RBC flow properties. Figure 3 highlights the results of the comparison of capillary RBC flow behavior before (pre) and after (post) dialysis by HDF. Overall, we saw an increase in pathological RBC shapes post-dialysis, as representatively shown in Figure 3A. Before the HDF, RBC flowed at equilibrium positions similar to healthy controls, representatively shown for patient 4 at *v* = 5 mm/s in the left panel of Figure 3B. Here, RBCs exhibited a similar number of off-centered slipper shapes and centered croissant-like shapes at this intermediate velocity regime. Hence, pronounced peaks in the distributions emerge at |y/W| = 0 and |y/W| ≈ 0.125, in good agreement with the distribution of a healthy control. However, post-dialysis, RBCs did not deform into the characteristic slipper shape at the same velocity but formed shapes that preferentially flowed in the channel center. Thus, the number of off-centered cells decreased while the number of centered RBCs increased, as indicated by the strong peaks at |y/W| = 0 and |y/W| = 0.125 in the right panel of Figure 3B. This distinct deviation in the cell’s *y*-distribution post-dialysis was observed for all patients (Figure 3C). Although both pre- and post-dialysis samples exhibited a larger number of pathological RBC shapes compared with healthy controls (Figure 3D), all patients showed a slight increase in the shape ratio post-dialysis.

### Evaluation of Stored Erythrocyte Concentrates

RBCs are the most common blood component used in transfusion medicine, with about 85 million units being transfused worldwide each year (1 unit ≃ 200 ml of packed RBCs) (García-Roa et al., 2017). After a blood donation, RBCs are separated from plasma, white blood cells, and platelets and stored at 4°C in appropriate buffers for up to 49 days (7 weeks). During this time, RBCs experience the so-called storage lesion, leading to metabolic changes (Kim-Shapiro et al., 2011), oxidative stress to proteins and lipids (Barshtein et al., 2011; Yoshida et al., 2019), cell shape (Doan et al., 2020) and oxygen uptake and release modifications (Chng et al., 2021). The morphological changes (Tinmouth et al., 2006; Moon et al., 2021; Park et al., 2021) and the decrease in deformability as a part of the storage lesion (Bennett-Guerrero et al., 2007; D’Alessandro et al., 2015; Matthews et al., 2015), result in an alteration of RBC flow behavior and a reduced microcapillary perfusion rate (Piety et al., 2021). However, easy and standardized tests that operate with small EC aliquots are still missing.

**FIGURE 4:**
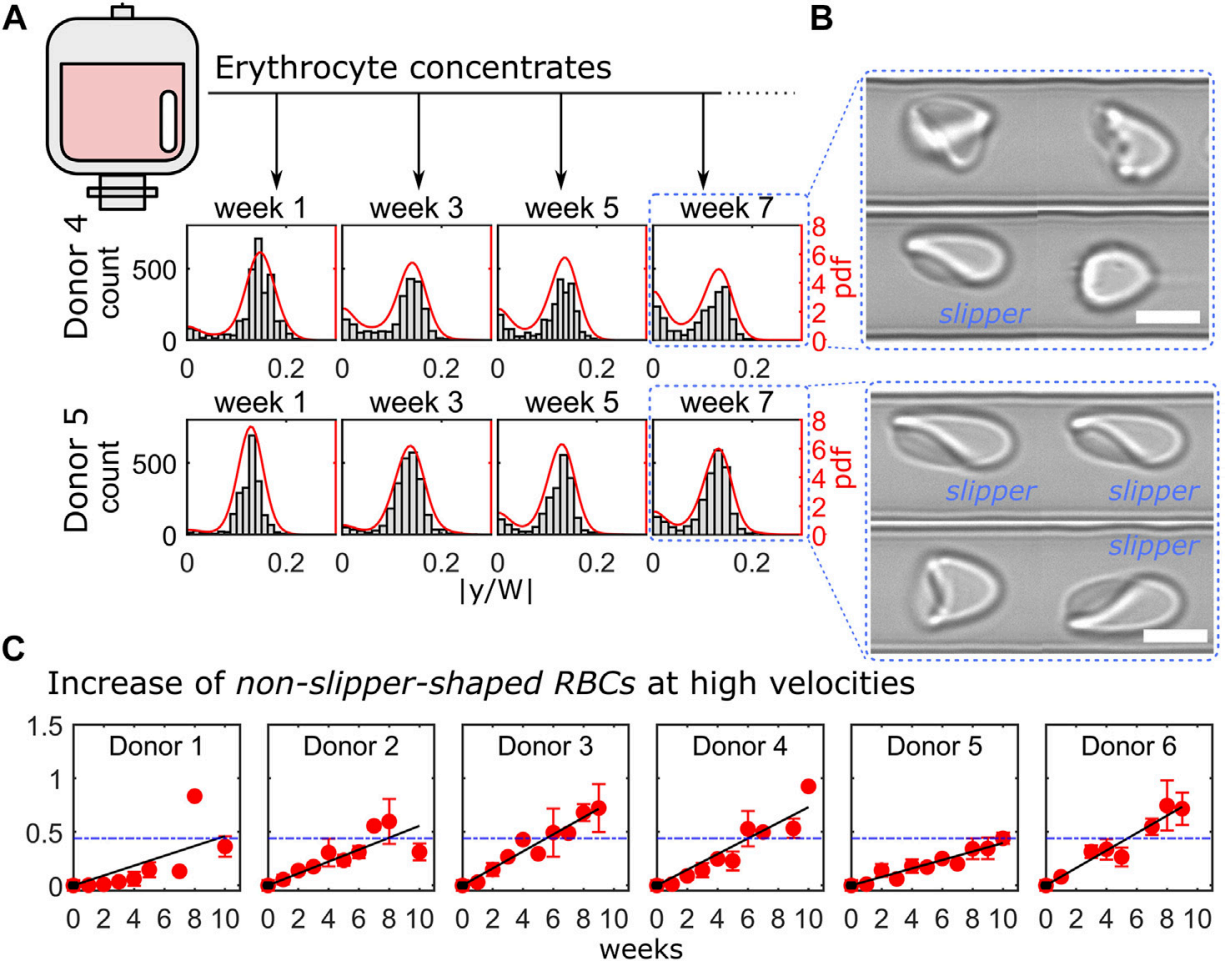
Cell shape characterization within 10 weeks of erythrocyte concentrate storage. **(A)** Representative histograms and probability density functions (pdfs) of the RBC y-positions for donor 4 (top) and donor 5 (bottom) at a velocity of 10 mm/s for four consecutive weeks. **(B)** Representative RBC shapes for both donors at week 7. Scale bars represent 5 µm. **(C)** Ratio between centered cell shapes and slipper-shaped RBCs for all donors as a function of time. Red symbols correspond to measurements’ average over a velocity range of 8–10 mm/s and black lines represent linear fits. Blue dashed horizontal lines indicate the mean value of the linear fits for all donors at week 6. The analysis presented was performed on in average 4847 cells per donor and time point (between 2213 and 9958 cells).

Here, we performed the microfluidic analysis of 6 ECs over a period of 10 weeks. We focused on the RBC flow behavior at high velocities, where cells preferentially deform into off-centered slippers. The distribution of the RBC equilibrium position at a velocity of 10 mm/s is representatively shown for donor 4 and donor 5 in Figure 4A for the four different weeks. For both donors, RBCs initially deformed into slippers at such high velocities, as indicated by the pronounced off-centered peaks in the pdfs and flow at the characteristic off-centered equilibrium position after the first week. As the ECs are stored, RBC deformability decreased (Matthews et al., 2015). Indeed, an increasing number of cells were not able to deform into stable slippers but exhibited various other shapes that flowed closer to the middle of the channel. Hence, we observed an increasing peak in the distributions at y = 0 in Figure 4A as storage time progressed. Representative RBC shapes for donors 4 and 5 at week 7 are shown in Figure 4B. However, this simultaneous decrease in the frequency of observed slipper-shaped RBCs and increase in other, more centered shapes is different for different donors. Here, we used this ratio of non-slippers to slippers at high velocities as a parameter to determine the change in RBC flow behavior of stored ECs over time. The increase of the fraction of non-slipper-shaped RBCs, normalized by the values for fresh controls, is shown for all investigated donors in Figure 4C. Here, measurements were combined over a velocity range of 8–10 mm/s, where slippers are commonly observed (Guckenberger et al., 2018; Recktenwald et al., 2022). Experimental data were fitted linearly over time. The average value of the fits for healthy blood donors at week 7 (49 days) is plotted as a horizontal blue line as a reference (expiration date of PAGGSM EC). While half of the examined samples (donors 3, 4, and 6) exceed this threshold approximately between weeks 5 and 6, this limit was reached for donors 1 and 2 roughly one or 2 weeks later. Remarkably, we found that the stored RBCs of donor 5 remained under this limit even after 9 weeks (63 days), highlighting the heterogeneity and donor-dependence of stored RBCs (Matthews et al., 2015; Islamzada et al., 2020).

## Discussion

Erysense enables a fast and unbiased assessment of RBC properties and shapes in capillary flow using a combination of microfluidic technologies and machine learning. The ability to adapt their shape to the vessel size and the flow conditions is crucial for microvascular RBC transport and is related to mechanical RBC properties, such as its bending rigidity and cytoplasm viscosity (Himbert et al., 2021). Our approach simulates capillary flow in rectangular microfluidic channels with channel dimensions slightly larger than the RBC diameter. In contrast to methods that rely on larger geometries (Piety et al., 2015; Alapan et al., 2016; Himbert et al., 2021), which result in a plethora of RBC shapes, or on constricted funnel-like channels (Matthews et al., 2015; Myrand-Lapierre et al., 2015; Rizzuto et al., 2021), the technique presented here allows us to assess the flow characteristic of stable RBC shape configurations in a broad velocity range. For deformable RBCs, the geometric asymmetry of the cross-section of the used channels results in the emergence of two dominating morphologies (centered croissants and off-centered slippers) depending on the flow velocity. The Erysense technology provides multiple modes to analyze the microscale RBC flow, enabling general applicability in various medical and clinical applications. In the study presented here, we evaluated the cell’s microscale flow behavior by focusing on the RBC lateral equilibrium position along the channel width as a characteristic parameter. Here, we found that impaired deformability, *e.g.*, of stored RBCs, results in a change in the RBC lateral distribution, especially at high velocities (Figures 2, 4). Additionally, employing machine learning algorithms enabled us to characterize pathological RBC morphologies under constant flow, similar to previous studies in stasis (Moon et al., 2021; Simionato et al., 2021; Song et al., 2021; Lamoureux et al., 2022). This allowed us to detect morphological changes, distinctive for a certain disease, such as the acanthocytes shown in Figure 2, or treatment (HDF, Figure 3). Changing the applied pressure drop and/or changing the viscosity of the surrounding fluid further provides opportunities to test the RBC flow behavior under a variation of the external shear stress. Besides these two operation modes (RBC lateral position and morphology), the presented method allowed us to further extract the single-cell projection area, RBC elongation based on the major and minor axis length of the cell, and to study dynamical RBC states (snaking, tumbling, swinging, and tank-treading motions) within the field of view, for potential future applications beyond the investigations shown here.

We anticipate that the Erysense technology will be a new approach in the field of *in vitro* diagnostics. It is a missing link between state-of-the-art diagnostics and practical medical experience since Erysense is capable of simulating and visualizing the capillary RBC flow of a particular patient *in vitro*. This approach will enable a novel diagnostic concept, a new kind of patient-related risk assessment and therapy compatible with point of care concepts (Kaestner and Bianchi, 2020). In Figures 2–4 we show examples of initial tests that represent several application fields that could highly benefit from Erysense: (i) diagnostics and theragnostics, here the example of NAS, (ii) dialysis optimization concepts, (iii) quality control and improved donor match in transfusion medicine.

(i) We showcased the use of our technology as a novel diagnostic measure to examine the microcapillary RBC flow of NAS patients. Their RBCs have different properties compared to healthy controls as already indicated in the disease group name. However, up to now, the classification of acanthocytes is difficult and prone to mistakes (Storch et al., 2005; Darras et al., 2021) leaving reliable diagnosis a challenge. Our first general approach based on the Erysense device already gives promising results comparing 4 healthy controls with 3 ChAc and 4 MLS patients. On the one hand, we could identify acanthocytes that exhibit distinct shapes in capillary flow compared to normocytes at low velocities. On the other hand, upon increase in the flow strength, we are further able to distinguish between healthy controls and pathological RBCs as their equilibrium flow positions are distinctively different. Further studies comprising a higher number of patients including differential diagnosis of other neurological diseases and testing the specificity of the method are compulsory before the RBC capillary flow can be considered as a functional diagnostic tool or biomarker for interventional studies for NAS.

(ii) We probed the influence of HDF on RBC flow properties and found severe effects. The investigation was limited to two time points, directly before and immediately after HDF, resembling a snapshot and requiring further analysis including measurements during HDF and the putative recovery of the RBCs after HDF. Correlating our measurements to previous studies (Georgatzakou et al., 2018), we are likely detecting oxidative-stress-driven damages and shape changes probably associated with the effective clearance of dialyzable natural antioxidant components, including uric acid, from the uremic plasma. However, Erysense bears the potential to accompany optimization processes, *e.g.*, membrane type or dialysis time of HDF and other forms of dialysis.

(iii) We evaluated storage-induced changes of ECs by assessing the variations for single cells flow at high velocities due to impaired deformability (Figure 4). We saw a considerable variety of the RBC properties over time for the different donors. Despite these clear effects on the cellular level, the association between EC storage time and clinical complications experienced by patients is unclear. Conflicting results were obtained in different clinical and prospective studies: some observed associations between storage time and patient post-transfusion recovery (Offner et al., 2002; Koch et al., 2020; Roussel et al., 2021), but several others found no differences (Lelubre and Vincent, 2013). However, it is known that after transfusion, up to 30% of transfused RBCs are removed from the body within 24 h (Luten et al., 2008). The remaining cells regain original functions due to a rejuvenation process occurring in the circulation to a certain extent (Barshtein et al., 2018). The reasons why a portion of cells is cleared from the body and how the rest recovers are not known. Additionally, clinicians are not always aware of how long transfused RBCs survive in the organism. This lack of knowledge limits decision-making about the volume and frequency of transfusions for each patient, which may be exposed to unnecessary risks of transfusion-associated complications. Further clinical studies making use of Erysense are not limited to solving these questions but may allow a prospective prediction of the expected behavior of a certain EC *in vivo,* providing a novel EC management. In general, cross-matched compatible blood is considered a safe product if blood units tests are negative regarding infectious diseases. However, this is a deception since donated blood might be far from being in a perfect condition or even suitable for a particular recipient as indicated in Figure 4. In fact, the functionality of RBC can be heavily restricted due to donor eligibility and health state, storage time, transportation, and handling. As a consequence, severe complications may arise at the time of transfusion or shortly thereafter (Barshtein et al., 2011, 2016).

We hypothesize that besides the examples provided in this study, further conditions, like diabetes (Tan et al., 2020), iron deficiency (Caruso et al., 2021), COVID-19 (Kubá nkov á et al., 2021), rheumatism (Giacomello et al., 1997), various RBC disorders and anemia (Huisjes et al., 2018; Pretini et al., 2019), inflammation-based diseases including sepsis (Piagnerelli et al., 2003; Bateman et al., 2017) result in subtle but systematic and reproducible adaptations of the RBC morphology in capillary flow. Additionally, the emerging field of drug delivery by RBC-based carriers, including nucleic acid delivery for gene therapy, requires a tool for quality control that accepts minimal sample volumes (Pelle and Kostevšek, 2021). Even pharmacological safety tests can likely be performed on RBCs under capillary flow. However, the highest application potential for the use of Erysense is expected in the clinical field as well as in the field of standard diagnostics. Serving as a pre- and postoperative monitoring system to test blood compatibility or thrombosis risk estimation, the technology can prospectively be used as standard equipment in any operating theatre and adjacent intensive care unit.

## Data Availability

The original contributions presented in the study are included in the article/Supplementary Material, further inquiries can be directed to the corresponding authors.
