# Supplementary material for: Erysense, a Lab-on-a-Chip-Based Point-of-Care Device to Evaluate Red Blood Cell Flow Properties With Multiple Clinical Applications
Source: Front Physiol. 2022 Apr 27;13:884690. doi: 10.3389/fphys.2022.884690 (PMC9091344; doi:10.3389/fphys.2022.884690)
Supplement: Supplementary file 1 [file DataSheet1.PDF]

Supplementary Material

1 SUPPLEMENTARY FIGURES AND TABLES

1.1 Figures

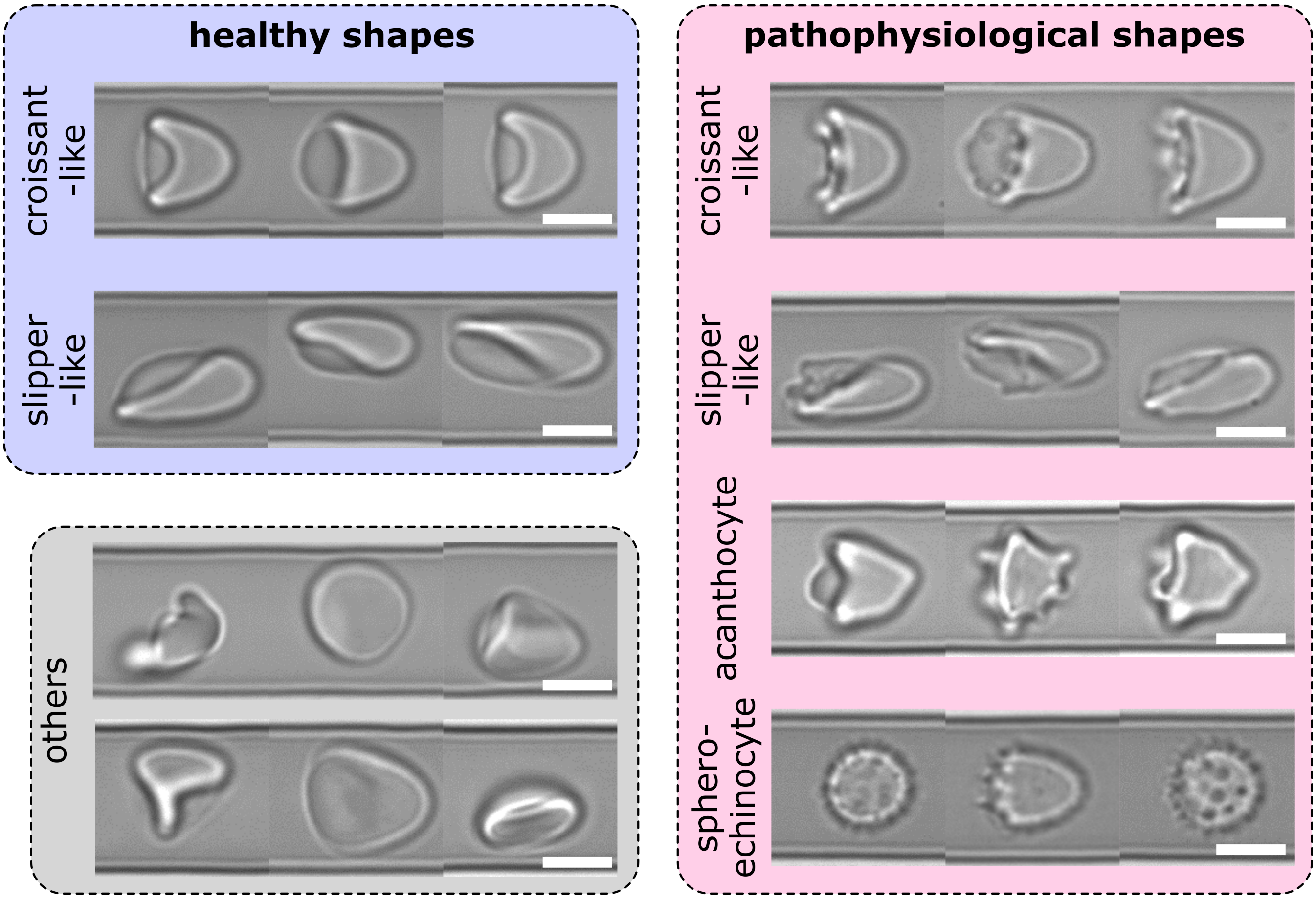

Figure S1. Characteristic RBC shapes used to train the CNN. RBC shapes are split into healthy and pathophysiological shapes and other shapes, which are not classified further. Scale bars represent a length of 5 µm

1.2 Tables

Table S1. Neuroacanthocytosis syndrome (NAS) patient overview. Chorea Acanthocytosis (ChAc) and McLeod Syndrome (MLS).

Subjects	Sex	Age (y)	Hematocrit (%)	Hemoglobin (g/dL)	RBC number (10^{12}/L)	Main clinical characteristics	Disease duration (y)	Acanthocyte Count (%)	Medication
MLS-1	male	59	43	16.0	4.82	myopathy	16	2	magnesium, St. John's wort extract, pumpkin seed preparation, PRN: pantoprazol
MLS-2	male	52	42	15.3	4.67	epilepsy, myopathy, peripheral neuropathy	41	32	levetiracetame 2 g/day, lamotrigine 400 mg/day, candesartan 8 mg/day, vitamin D, PRN: methylprednisolone
MLS-3	male	53	45	16.2	5.17	neuropathy, myopathy	19	24	No medication
MLS-4	male	56	47	16.4	5.18	kardiomyopathy, neuropathy	5	7	bisoprolol, eplerenone, apixaban, torasemide
ChAc-1	male	36	46	16.2	5.40	drug-resistant epilepsy, mild chorea, tics, cognitive impairment, peripheral neuropathy, myopathy	13	4	lacosamide 550 mg/day, zonisamide 300 mg/day, perampanel 4 mg/day, vitamin D, PRN: lorazepam/midazolam
ChAc-2	male	31	43	15.7	4.91	drug-resistant epilepsy, mild chorea, tics, cognitive impairment, irritability, anxiety, depression, psychosis	17	11	zonisamide 300 mg/d, aripiprazole 10 mg/d
ChAc-3	male	44	41	15.2	4.96	epilepsy, feeding dystonia, orofacial dyskinesia, chorea, peripheral neuropathy, myopathy, impulse control disorder	14	5	quetiapine 300 mg/d, levetiracetam 500 mg/d, lamotrigine 200 mg/d, melperone 100 mg/d

Table S2. Dialysis patient overview. Hemodiafiltration (HDF) and hemodialysis (HD). HDF pre and HDF post correspond to re-infusion processes pre-expansion and post-replacement, respectively.

Patient	Sex	Age (y)	Dialysis	Therapy duration (y)	Clinical characteristics
Patient 1	male	60	HDF post	30	hypertensive nephropathy
Patient 2	female	38	HD	17	reflux nephropathy
Patient 3	male	74	HDF pre	5	IgA nephropathy
Patient 4	male	72	HDF post	12	Urosepsis

Table S3. Overview of the used layers in the CNN.

Layer	Kernel size (px^2)	Subimage size (px^2)
input layer	–	140×160
conv. layer 1	3×3	140×160
reLU layer	–	140×160
max pooling layer	2×2	70×80
conv. layer 2	3×3	70×80
reLU layer	–	70×80
max pooling layer	2×2	35×40
conv. layer 3	3×3	35×40
reLU layer	–	35×40
max pooling layer	2×2	17×20
output layer	–	1×11
